# Study on rock fracture mechanism and hydraulic fracturing propagation law of heterogeneous tight sandstone reservoir

**DOI:** 10.1371/journal.pone.0303251

**Published:** 2024-08-02

**Authors:** Huan Zhao, Wei Li, Meng Cai, Biao Ma, Xiaorui Xie, Linhao Zou, Yapeng Liu

**Affiliations:** 1 Northeast Petroleum University, College of Petroleum Engineering, Daqing, China; 2 Daqing Oilfield Co Ltd, Daqing, China; 3 National Key Laboratory of Continental Shale Oil, Daqing, China; 4 National Engineering Research Center of Oil & Gas Drilling and Completion Technology, Beijing, China; 5 Research Institute of Oil Production Engineering of Daqing Oilfield Co Ltd, Daqing, China; 6 Heilongjiang Provincial Key Laboratory of Oil and Gas Reservoir Stimulation, Daqing, China; China University of Mining and Technology, CHINA

## Abstract

Hydraulic fracturing technology is an effective way to develop tight sandstone reservoirs with low porosity and permeability. The tight sandstone reservoir is heterogeneous and the heterogeneity characteristics has an important influence on fracture propagation. To investigate hydraulic fracture performance in heterogeneous tight reservoir, the X-ray diffraction experiments are carried out, the Weibull distribution method and finite element method are applied to establish the uniaxial compression model and the hydraulic fracture propagation model of heterogeneous tight sandstone. Meanwhile, the sensitivity of different heterogeneity characterization factors and the multi-fracture propagation mechanism during hydraulic fracture propagation is analyzed. The results indicate that the pressure transfer in the heterogeneous reservoir is non-uniform, showing a multi-point initiation fracture mode. For different heterogeneity characterization factors, the heterogeneity characteristics based on elastic modulus are the most sensitive. The multi-fracture propagation of heterogeneous tight sandstone reservoir is different from that of homogeneous reservoir, the fracture propagation morphology is more complex. With the increase of stress difference, the fracture propagation length increases. With the increase of injection rate, the fracture propagation length increases. With the increase of cluster spacing, the propagation length of multiple fractures tends to propagate evenly. This study clarifies the influence of heterogeneity on fracture propagation and provides some guidance for fracturing optimization of tight sandstone reservoirs.

## Introduction

Tight sandstone reservoirs are widely distributed and have great potential, which are important resources for future development. Tight sandstone reservoirs have the characteristics of low permeability and low porosity. At present, staged multi-cluster fracturing of horizontal wells is an effective measure to develop tight sandstone reservoirs [1, 2]. The horizontal well staged multi-cluster fracturing technology, which is the important stimulation measures for the development of unconventional oil and gas resources, can complete the propagation of multiple hydraulic fractures through pumping fracturing fluid, realize the effective development of tight sandstone reservoirs and reduce the construction cost [3–5]. Meanwhile, the tight sandstone reservoir has obvious heterogeneity, which refers to the comprehensive influence of sedimentation, diagenesis and late tectonic action in the long geological history of oil and gas reservoirs, so that the spatial distribution of reservoirs and various internal attributes have extremely uneven changes [6–8]. It holds important implications in the fracture propagation and the development of sandstone reservoirs.

With regard to reservoir heterogeneity, a large number of researches have been carried out [9]. Andrew D.Mill proposed that the geological properties of reservoirs are unevenly distributed in space firstly. It mainly refers to the existence of different degrees of geological heterogeneity in the reservoir, including the changes of formation thickness, porosity, permeability, porosity distribution, lithology and stratigraphic structure in different locations. Reservoir heterogeneity can be classified according to different research scales. Pettijohn [10] proposed that the reservoir heterogeneity can be divided into layer level, sand body level, bedding level, lamina level and pore level through the macro and micro analysis of the small layer. Liu [11] analyzed the microscopic heterogeneity of Chang 3 sandstone reservoir in Zhenbei area, and concluded that it had a great relationship with diagenesis and microscopic pore size of reservoir rock. Wang [12] used the variogram to analyze the heterogeneity, which can truly reflect the spatial distribution characteristics of geological variables. Guo [13] studied the microscopic heterogeneity of marine shale reservoirs in the lower part of Longmaxi Formation and Wufeng Formation in Chongqing, and concluded that heterogeneity affects the microscopic pore types, gas reservoir distribution and gas reservoir migration capacity of marine reservoirs. In the study of the heterogeneity of tight sandstone reservoirs in the Shanxi Formation of the Ordos Basin, Lu [14] used the combination of Matlab program mathematical methods to characterize the pore system of the microscopic heterogeneity of tight reservoirs. Li [15] combined with CT scanning technology to analyze the evolution law of fracture initiation and propagation in low permeability reservoirs under uniaxial compression test, and analyzed the relationship between heterogeneity characteristics and fracture morphology of low permeability reservoirs.

The multi-fracture propagation of horizontal wells is affected by many factors, and the stress interference between multi-fractures in reservoirs. In order to study the law of multi-fracture propagation, scholars have adopted a series of methods, including finite element method [16, 17] discrete element method [18, 19], displacement discontinuity method [20, 21] experimental analysis method [22, 23] and so on. The research results show that there is stress interference between multiple fractures in horizontal wells, and the mechanical characteristics of the reservoir, injection rate and perforation parameters have obvious influence on the propagation morphology of multiple fractures. However, at present, the research is mainly carried out on the multi-fracture propagation of horizontal wells in homogeneous reservoirs, and the heterogeneity characteristics of reservoirs are less considered. The characteristics of reservoir heterogeneity have a very important influence on the propagation of multi-fractures in hydraulic fracturing. The introduction method of rock heterogeneity is an important work to quantitatively characterize the degree of rock heterogeneity. A reasonable introduction method is very important for generating a numerical model of rock heterogeneity that conforms to the characteristics of real materials.

In this paper, the reservoir heterogeneity model is established by using Weibull distribution random characterization method. The rock fracture modes of homogeneous model and heterogeneous model are compared and analyzed. The sensitivity of fracture propagation law to different heterogeneity characterization factors during hydraulic fracture propagation is analyzed. A multi-fracture propagation model of heterogeneous reservoir is established. The influence of stress difference, injection rate and fracture spacing on hydraulic fracture propagation is analyzed, and the multi-fracture propagation law of heterogeneous tight sandstone reservoir is clarified. The research will provide a basis for hydraulic fracturing design of tight sandstone reservoirs.

## Reservoir heterogeneity characteristics and characterization

The reservoir has the characteristics of heterogeneity, which has an important influence on the fracture propagation morphology during hydraulic fracturing. The heterogeneity of the reservoir is mainly manifested in the non-uniform distribution of different mineral particles in the rock and the distribution of natural fractures in the reservoir. In the analysis of hydraulic fracturing fracture propagation, it is very important to consider the heterogeneity of the reservoir.

### The characteristics of rock heterogeneity

**Fig 1:**
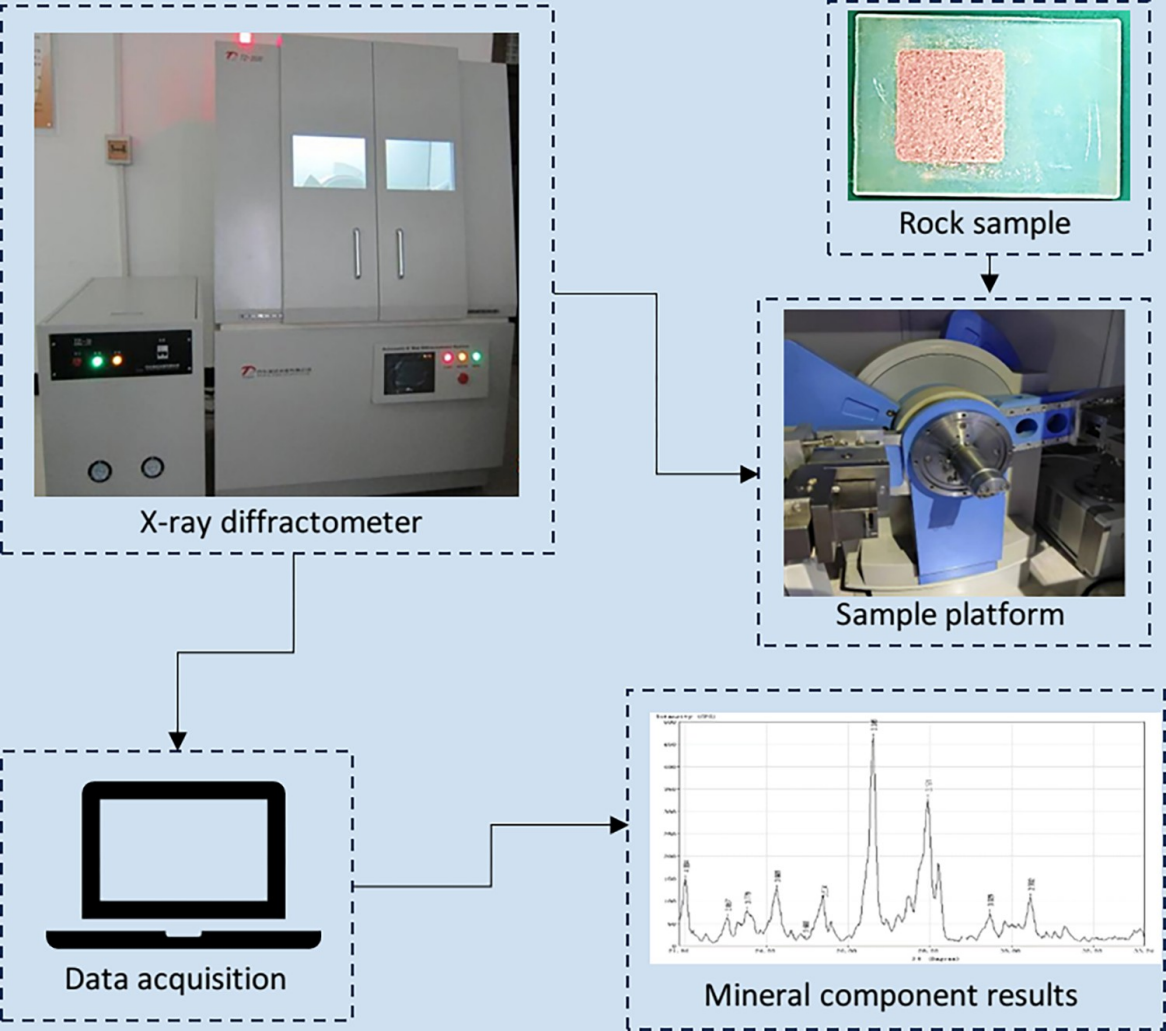
XRD diffraction experimental instrument and experimental results.

The change of relative content of minerals will make the rock show different mechanical properties. The X-ray diffraction experiment of block A is carried out to test the mineral composition of block A. Brittle minerals and clay minerals are related to the effect of hydraulic fracturing, and other minerals such as pyrite and siderite affect the internal filling state of microcracks. The core mineral composition test experiments of two small layers of Z-1 sub-section were carried out by X-ray diffractometer which belongs to National Engineering Research Center of Oil & Gas Drilling and Completion Technology, as shown in Fig 1.

The determination shows that the relative content of mineral components in different lithic sandstones is different, including brittle minerals such as feldspar, quartz, calcite, and anhydrite. The mineral composition of sample A includes 5.2% anhydrite, 37% quartz, 49% plagioclase and 8.8% calcite. The mineral composition of sample B includes 7.3% anhydrite, 45% quartz, 41% plagioclase and 6.7% calcite. It can be seen that there are some differences in the mineral composition of sandstone at different depths of the same small layer, which proves that the reservoir has the characteristics of horizontal and vertical heterogeneity.

According to the results of X-ray diffraction experiments, it can be known that different mineral compositions and contents in rocks, huge pressure and temperature changes in the formation process, and the influence of different geological historical periods will all have an impact on its internal structure, resulting in a variety of heterogeneous characteristics of reservoir physical properties.

### Reservoir heterogeneity characterization and heterogeneity model

Rock heterogeneity characterization methods based on statistical theory are widely used by scholars at home and abroad [24, 25]. In order to characterize the heterogeneity of rock mechanical parameters, scholar proposed to use Weibull distribution to characterize its heterogeneity characteristics [26, 27]. It is assumed that the rock mechanical parameters are random variables that obey the Weber distribution, and the probability density distribution function is:

$$f(x,m,n)=\{\begin{array}{c} \frac{m}{n}{\left(\frac{x}{n}\right)}^{m-1}e^{-{\left(\frac{x}{n}\right)}^{n}}\\ 0 \end{array}\begin{array}{cc} & x\geq 0\\ & x<0 \end{array}$$ (1)

where x represents a random variable; m represents the dimensionless shape parameter, determines its distribution function properties, reflects the degree of rock homogeneity. when m > 1, it represents the heterogeneity of hard small deformation material, and when m<1, it represents the heterogeneity of soft large deformation material, and the shape parameter m is smaller, the rock heterogeneity is stronger. n is a proportional parameter related to the average value of rock parameters.

When the heterogeneous finite element model is established, the Numpy library can be used to characterize the heterogeneity of the basic parameters of reservoir rock. Python is used to write a program to extract and rewrite the material properties, and generate a Weibull distribution code with the basic physical and mechanical parameters of the specified heterogeneous rock. By compiling the function library, the Weibull distribution random number is generated by traversing each unit number, and the rock data is simulated to generate a fracture propagation model considering rock heterogeneity. By using the K code to introduce heterogeneity, the value of which is the product of m and the numpy.random.weibull () function to generate random numbers. The general program code is shown in Table 1.

**Table 1:** Code table of Weibull distribution.

Code	Code action
from abaqus import *	Import model
from abaqusConstants import *	Import model
import numpy	Import numpy function library
num_elem = getInput(’number of element’):	Input the number of generated grids
for k in range(int(num_elem))	Traversing grid elements
K = m*numpy.random.weibull(shape,int(num_elem))	Random parameters conforming to Weber distribution

### The effect of heterogeneity on rock mechanical properties

The rock is a material containing micro-cracks, micro-holes and other meso-defects, that is, the basic physical and mechanical parameters of each micro-element of the rock are not the same, and the physical and mechanical parameters of each micro-element obey the Weibull distribution. Based on this theory, the rock failure mechanism and strength problem considering the heterogeneity of the micro-element are studied. The standard rock model is established and the uniaxial compression numerical simulation is carried out. The stress of the rock in the underground is simulated by applying a certain pressure, so as to understand its strength and deformation characteristics.

The interaction between rock particles of uniaxial compression is simulated by embedding cohesive elements [28, 29]. The rock model is based on the Drucker-Prager constitutive model and the embedded model is based on BK (Benzeggagh-Kenane) model.

The Drucker-Prager constitutive model is as follows,

F=t−ptanβ−d=0 (2)

$$t=\frac{1}{2}q[1+\frac{1}{K}-(1-\frac{1}{K}){\left(\frac{r}{q}\right)}^{2}],\;d=(1-\frac{1}{3}\beta )\sigma_{c}$$ (3)

Where, *β* is the slope of the linear yield surface on the *p*-*t* stress surface is generally related to the internal friction Angle of the material. *d* is the cohesion of the material. *K* is the ratio of uniaxial tensile yield stress to uniaxial compressive yield stress.

The quadratic nominal stress criterion was selected as the initial damage criterion, which states that damage starts when the ratio of the square of the nominal stress in each direction is equal to 1, which can be expressed as:

$${\left\{\frac{\langle t_{n}\rangle }{t_{n}^{0}}\right\}}^{2}+{\left\{\frac{t_{s}}{t_{s}^{0}}\right\}}^{2}=1,$$ (4)

where *t*_n_^0^ and *t*_s_^0^ are the peak nominal stresses when the normal and tangential deformations are perpendicular to the interface, respectively, *t*_n_ and *t*_s_ are the normal and tangential stress components, respectively, and the 〈 〉 brackets indicate that damage is not caused by purely compressive deformation or the stress conditions.

BK model is as follows,

$$G_{\mathrm{TC}}=G_{\mathrm{IC}}+(G_{\mathrm{IIC}}-G_{\mathrm{IC}}){\left(\frac{G_{\mathrm{II}}}{G_{T}}\right)}^{\eta }$$ (5)

$$G_{T}=G_{I}+G_{\mathrm{II}}$$ (6)

Where, *G*_I_ is the energy release rate of type I, *G*_II_ is the energy release rate of type II, *G*_IC_ is composite fracture toughness of type I, *G*_IIC_ is composite fracture toughness of type II, *G*_T_ is the total energy release rate, *G*_TC_ is composite fracture toughness, *η* is the BK criterion constant.

**Fig 2:**
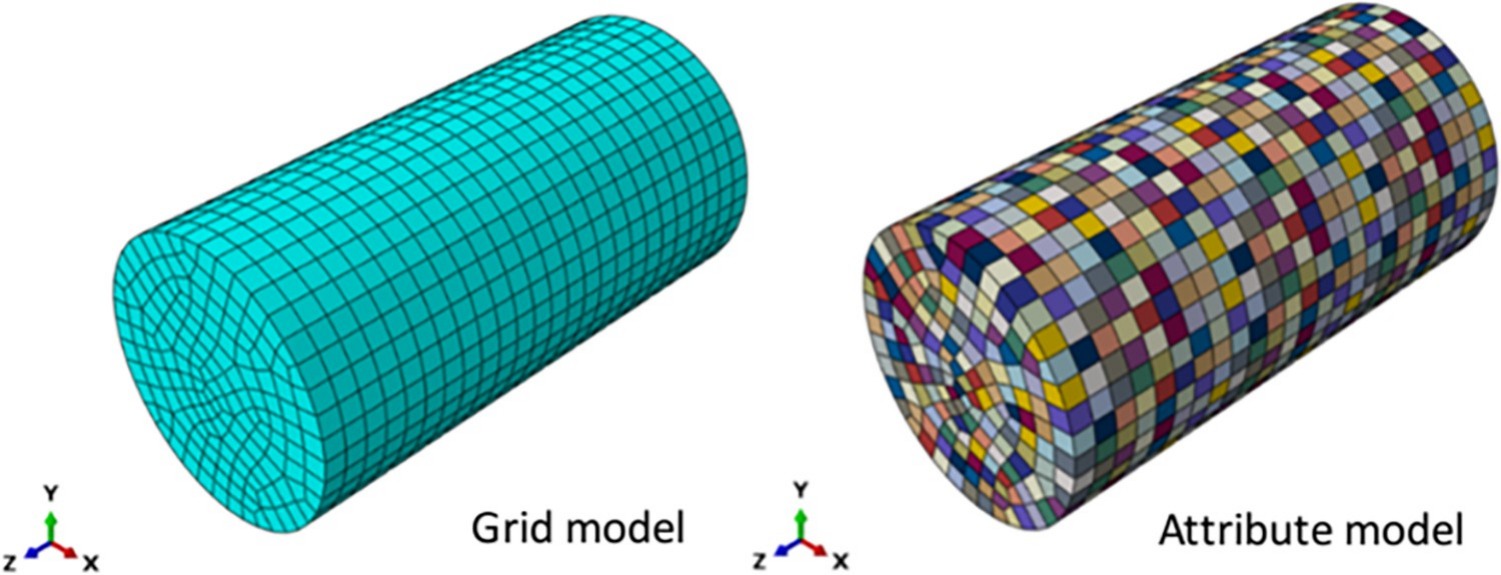
Numerical model of uniaxial compression.

Through the numerical simulation of heterogeneous uniaxial compression, the influence of rock parameter heterogeneity on mechanical properties is analyzed. According to this condition, the numerical simulation scheme is determined. The size of the simulated rock sample is Φ25mm×50mm. The model grid adopts the structured division technology. The rock matrix element type is selected as the three-dimensional stress. The unit defaults to select the reduced integral unit. The unit type is C3D8R, and the grid shape is hexahedron. In the model, a total of 3975 unit grids are divided. M represents the heterogeneity characterized by elastic modulus. The rock heterogeneity models with m = 2(strong heterogeneity), 6(moderate heterogeneity), and 10(weak heterogeneity) were established. In the models generated by using heterogeneous rock models, the elastic modulus parameters of mesoscale elements followed Weibull distribution, as shown in Fig 2.

**Fig 3:**
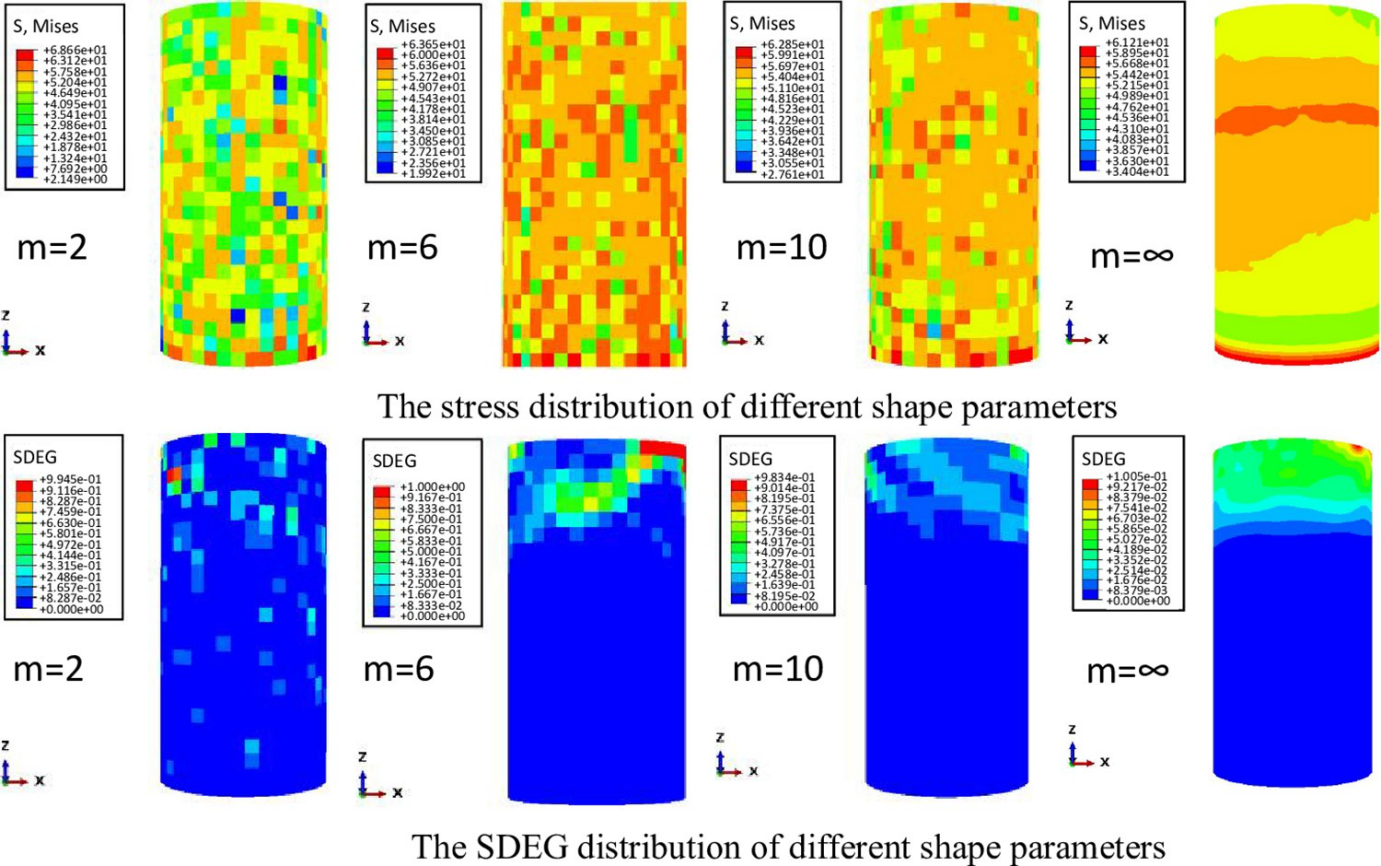
Simulation results of different heterogeneity models.

The simulated loading of uniaxial compression test is divided into two steps. The first step is to set the upper and lower boundary conditions, and the second step is to use displacement loading, and the displacement loading is 0.5mm. The stress distribution and SDEG loss distribution of different heterogeneity were plotted, as shown in Fig 3.

Comparing and analyzing the above stress distribution nephogram, it can be seen that the stronger the heterogeneity of elastic modulus is, the larger the range of rock stress is, and the greater the mechanical difference at different positions is. The stress distribution of homogeneous rocks is more uniform, and the fracturing point is concentrated in one position, while the stress distribution of heterogeneous rocks is uneven, and the stress concentration appears at multiple points, and the fracture may occur at multiple points.

The SDEG is the stiffness degradation variable during the loading process of the element. The variable describes the influence of the gradual decrease of the stiffness of the material during the loading process. When SDEG > 0.9, the element will be destroyed. By analyzing the distribution of stiffness degradation parameter SDEG in different degrees of heterogeneity models, it can be seen that the stronger the heterogeneity, the more dispersed the crushing unit, and the easier it is to fracture at multiple points. Therefore, considering the heterogeneity of the reservoir is very important for the analysis of rock initiation and fracture propagation.

## Hydraulic fracture propagation model considering reservoir mineral heterogeneity

### The propagation law of single cluster hydraulic fracture in heterogeneous reservoir

**Fig 4:**
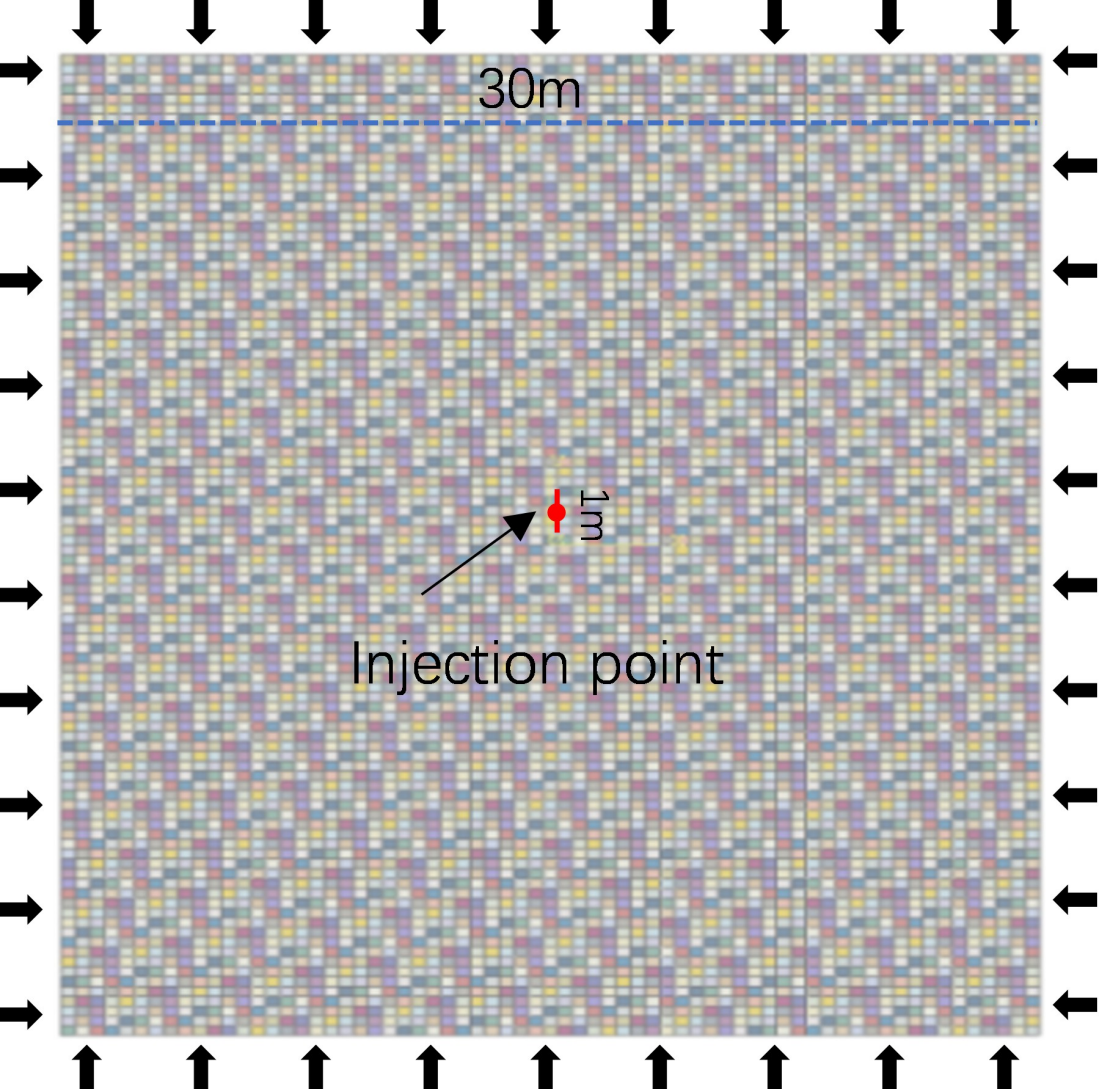
Heterogeneous rock model of single fracture.

Based on the assumption that the reservoir mechanical parameters obey the Weber distribution, different shape parameters are used to describe the reservoirs with different degrees of heterogeneity, and a single cluster hydraulic fracture propagation model is established. When m = 2, the model represents strong heterogeneity. When m = 6, the model represents moderate heterogeneity. When m = 10, the model represents weak heterogeneity. Considering the heterogeneity of reservoir rock from three aspects of elastic modulus, Poisson ’s ratio and tensile strength, the influence of heterogeneity characterized by different rock mechanical parameters on the length of hydraulic fracture is studied. According to different heterogeneous conditions, the hydraulic fracture propagation length is compared with the results of the homogeneous model, and the sensitivity of different heterogeneity characteristics to the influence of hydraulic fracture length is analyzed. The single cluster hydraulic fracture propagation model of different heterogeneity is shown in Fig 4. The specific parameters of the model are shown in Table 2.

**Table 2:** Basic parameters of heterogeneous sandstone reservoir model.

	Parameter	Value	Unit
Reservoir rock	Shape parameter	2,6,10, ∞	
	Elastic modulus	15	GPa
	Poisson ratio	0.25	
	Effective permeability	1×10^−7^	μm^2^
	Porosity	5	%
	Tensile strength	6	MPa
	Tensile fracture energy	30000	N/m
	BK criterion coefficient	2.284	
	Formation filtration coefficient	1×10^−14^	m/(Pa·s)
Fracturing fluid	Fluid specific gravity	9800	N/m^3^
	Fluid viscosity	0.001	Pa·s
	Injection rates	0.002	m^3^/s

**Fig 5:**
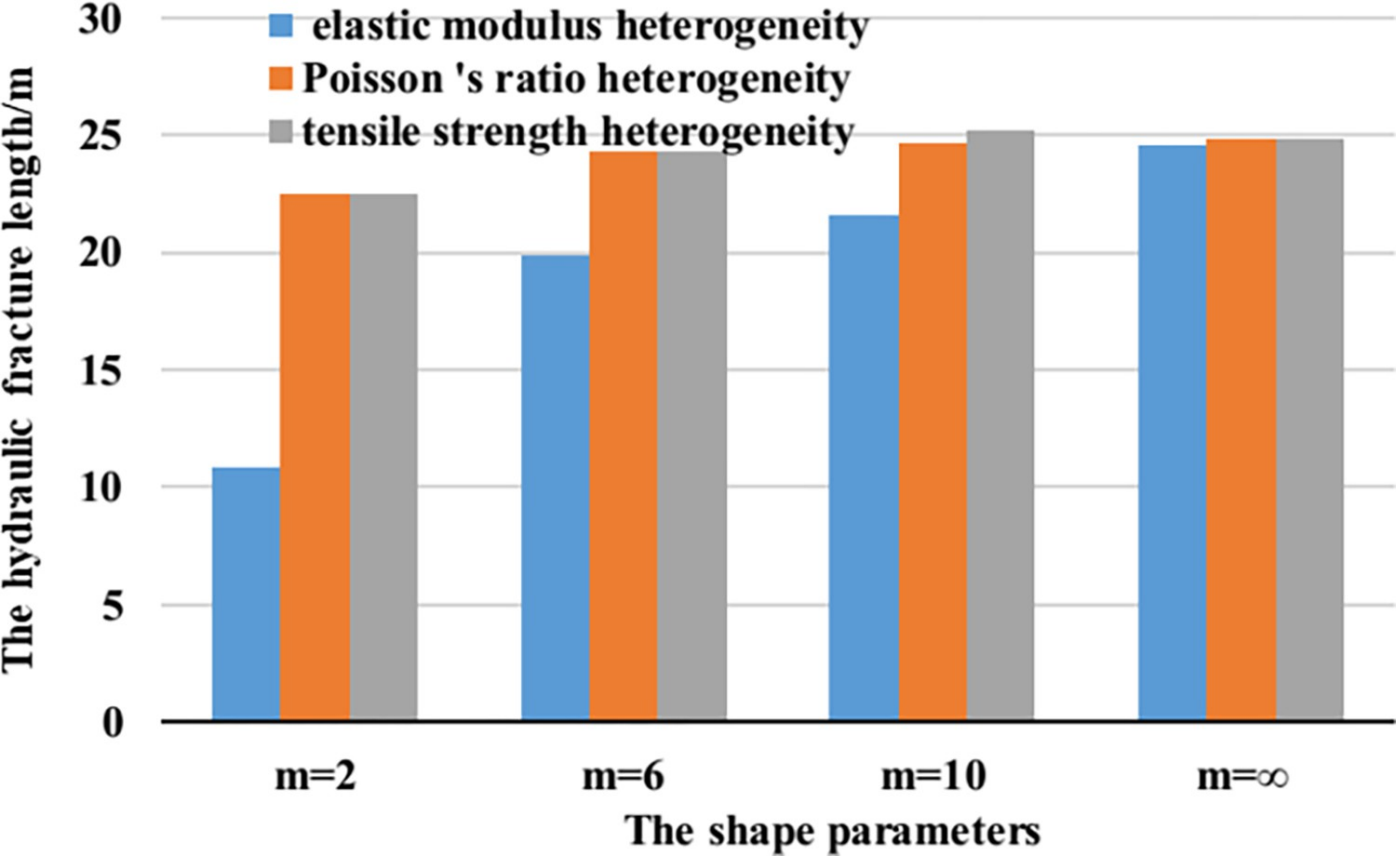
Comparison of different heterogeneity characterization methods.

The hydraulic fracture propagation length comparison diagram of different heterogeneity characterization methods is drawn, as shown in Fig 5. It can be seen from the diagram that the hydraulic fracture length of homogeneous reservoir (m = ∞) is 24.60 m in the reservoir characterized by elastic modulus heterogeneity. The hydraulic fracture length of the weakly heterogeneous reservoir (m = 10) is 21.62 m. The hydraulic fracture length of medium heterogeneity reservoir m = 6) is 19.85 m. The hydraulic fracture length of the strong heterogeneity reservoir is 10.83 m, and the fracture length changes obviously, with a variation range of 55.97%. In the reservoir characterized by Poisson ’s ratio heterogeneity, the hydraulic fracture length of homogeneous reservoir (m = ∞) is 24.85 m; the hydraulic fracture length of the weakly heterogeneous reservoir (m = 10) is 24.61 m. The hydraulic fracture length of medium heterogeneity reservoir (m = 6) is 24.31 m. The hydraulic fracture length of strong heterogeneity reservoir (m = 2) is 22.52 m, and the variation range is 9.38%. In the reservoir characterized by tensile strength heterogeneity, the hydraulic fracture length of homogeneous reservoir (m = ∞) is 24.85 m; the hydraulic fracture length of the weakly heterogeneous reservoir (m = 10) is 25.20 m. The length of medium heterogeneity hydraulic fracture is 24.31 m. The length of strong heterogeneity hydraulic fracture is 22.52 m, and the variation range is 9.38%.

By comparing and analyzing the simulation results of heterogeneity characterization of different rock mechanics parameters, it can be concluded that the heterogeneity characterized by elastic modulus has a great influence on the length of hydraulic fracture, and the heterogeneity of Poisson ’s ratio and tensile strength has no effect on the length of hydraulic fracture, which is consistent with Zeng Qingdong ’s research [30]. This is because the spatial distribution of rock elastic modulus in strongly heterogeneous reservoirs is often uneven, and there is a large spatial heterogeneity. Therefore, in the process of hydraulic fracturing, when the fracture encounters the area with higher elastic modulus, it will encounter greater resistance, which will limit the fracture propagation and lead to shorter fracture length. On the contrary, when the fracture encounters the area with lower elastic modulus, the fracture propagation speed will be accelerated due to the smaller resistance of the area. However, due to the heterogeneity of rock materials, these areas with lower elastic modulus may be scattered and cannot support longer fracture propagation, so the fracture length will also be limited.

### Hydraulic fracture propagation model of horizontal well in tight sandstone heterogeneous reservoir

In the process of hydraulic fracturing, the fracturing fluid, the formation stress and pressure difference work together to form hydraulic fractures. The propagation of fractures will affect the deformation of the rock skeleton by changing the pore pressure of the formation and the stress state of the rock, and in turn affect the propagation of fractures. In heterogeneous reservoirs, the propagation behavior of multiple hydraulic fracture clusters is more complicated. In the analysis of this situation, it can be based on the following assumptions [31, 32], (1) the reservoir stress direction is flat with the wellbore trajectory; (2) The rock layer is a saturated porous medium without isotropic and plastic effects, and there is no compression deformation between particles; (3) Hydraulic fractures are filled with incompressible fracturing fluid; (4) Hydraulic fracture propagation is a quasi-static process, without considering the influence of temperature.

**Fig 6:**
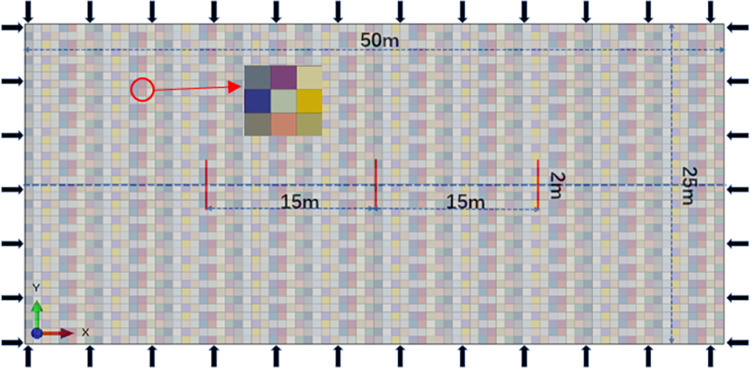
Two-dimensional finite element geometric model and its model parameter non-homogeneous distribution.

**Fig 7:**
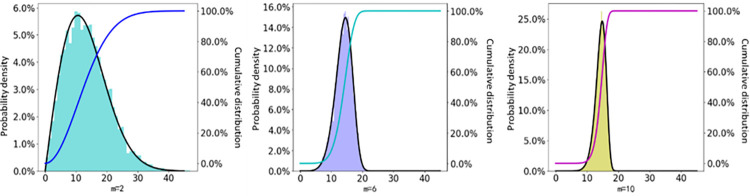
Statistical figure of elastic modulus under different heterogeneity conditions.

The problem of three-dimensional propagation of hydraulic fractures is simplified as a two-dimensional stress-strain problem perpendicular to the fracture height. On this basis, a two-dimensional hydraulic fracture propagation model is established. The spacing of hydraulic fracturing clusters is 15 m. Based on the heterogeneity of elastic modulus, a two-dimensional horizontal well three-cluster hydraulic fracture propagation model is established. The geometric model is shown in Fig 6, and the elastic modulus heterogeneity characterization method is used to establish multi-fracture interference hydraulic fracture propagation models with different heterogeneity degrees as shown in Fig 7. The specific parameters of the model are shown in Table 2.

### The influence of different heterogeneity characteristics on hydraulic fracture propagation

**Fig 8:**
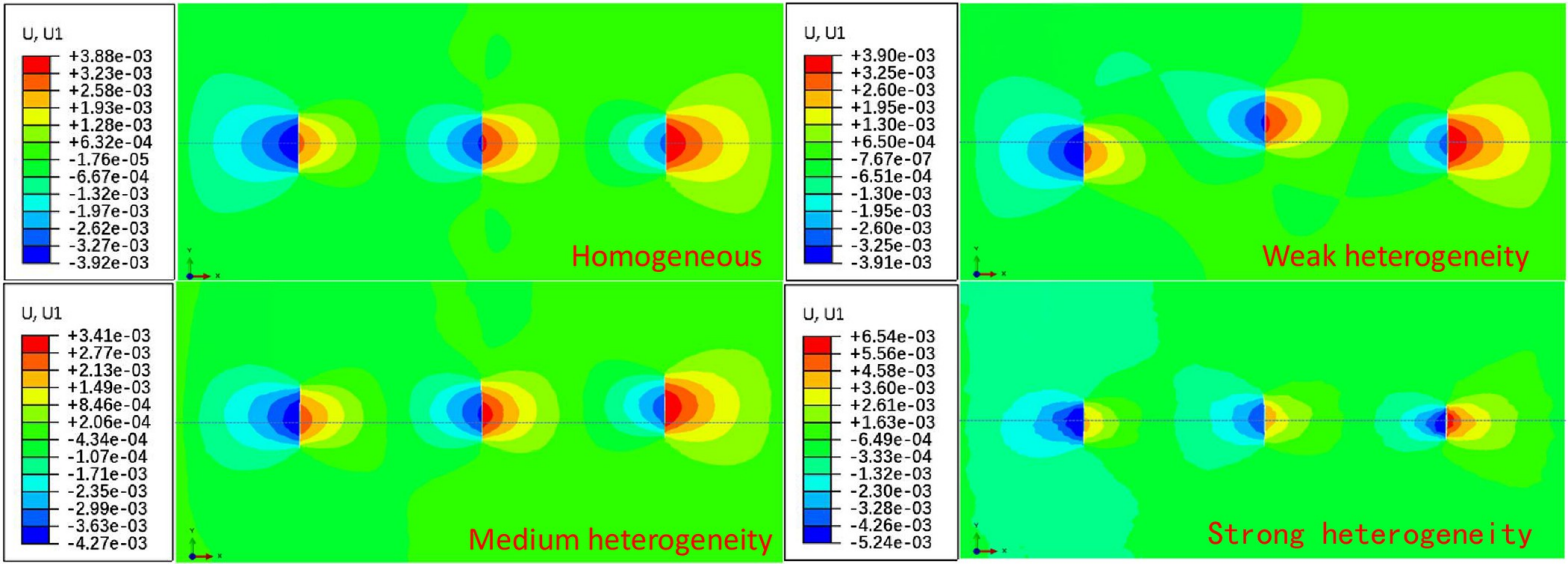
Horizontal displacement distribution of three clusters of hydraulic fractures propagation morphology in homogeneous and heterogeneous reservoirs.

The propagation morphology of three clusters of hydraulic fractures are plotted in homogeneous and heterogeneous reservoirs. As shown in Fig 8, asymmetric propagation is shown in heterogeneous reservoirs with different degrees of heterogeneity, while symmetrical propagation is shown in homogeneous reservoirs along the direction of maximum horizontal stress. When the heterogeneity is weak, the vertical propagation range of hydraulic fracturing is relatively large. When the heterogeneity is strong, the vertical propagation range of fractures is small, and the average fracture width increases with the increase of heterogeneity.

Using the finite element unit number to query the elastic modulus distribution of the propagation path, it can be known that the direction of hydraulic fracture propagation is the same as that with high elastic modulus. This is because under the same Poisson ’s ratio, the critical fracture energy required for reservoir rupture with higher elastic modulus is smaller. Therefore, in heterogeneous reservoirs, hydraulic fractures show an asymmetric propagation pattern, and tend to extend to the formation with higher elastic modulus to the most brittle.

**Fig 9:**
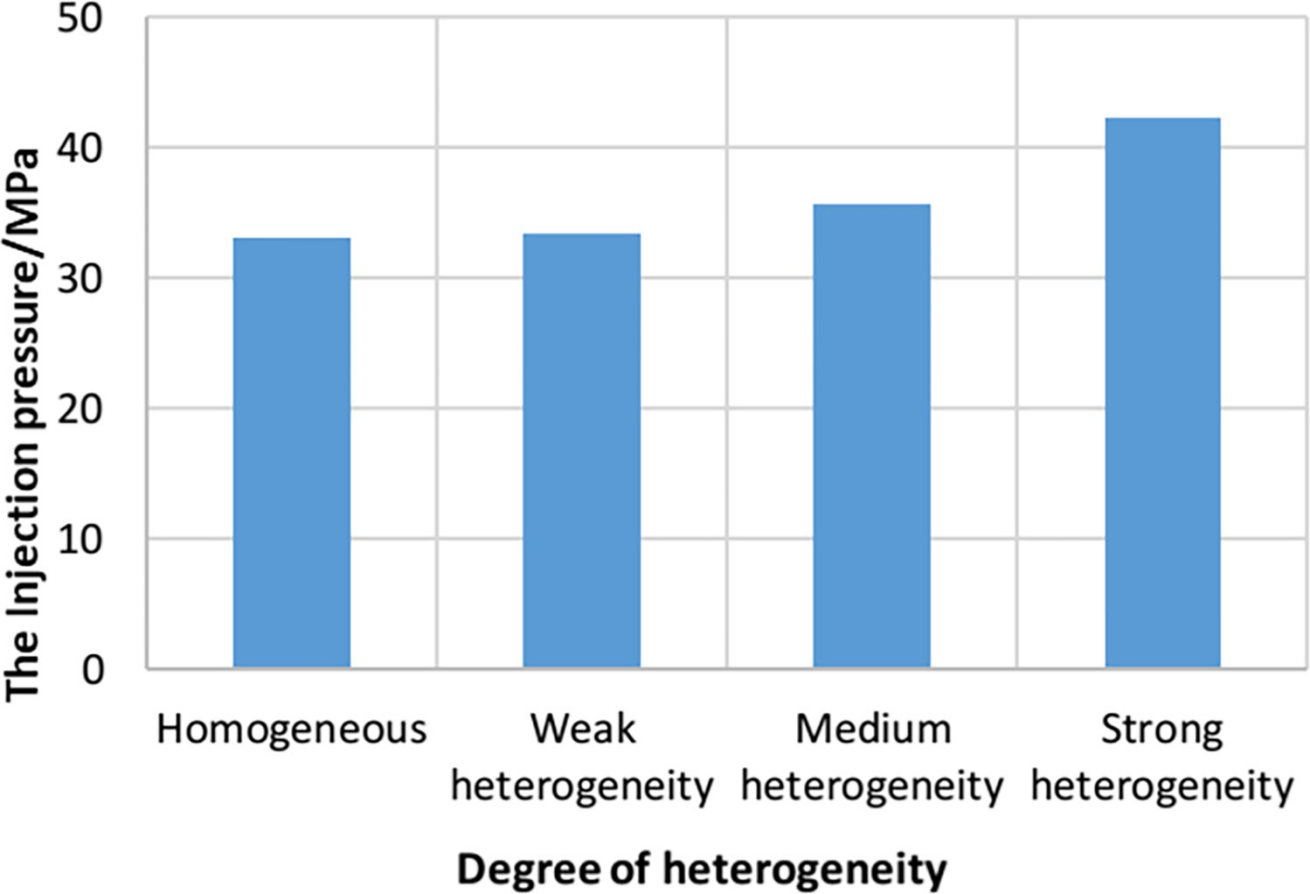
Injection pressure curves of intermediate hydraulic fractures in different heterogeneity models.

As shown in Fig 9, when the three hydraulic fractures propagate in heterogeneous reservoirs and homogeneous reservoirs, the fracture pressure curves of the middle hydraulic fractures show different changes. Compared with homogeneous reservoirs, the propagation pressure of hydraulic fractures in heterogeneous reservoirs is relatively high and fluctuates greatly. This is consistent with the conclusion of researcher Huang [33]. The reason for this result is that there are different defects such as mineral particles and pores in the rock. These defects cause different heterogeneity (such as different distribution of elastic modulus), change the stress concentration phenomenon in the rock, and lead to the increase of injection pressure in the fracture.

## Analysis of hydraulic fracture propagation law of horizontal wells considering reservoir mineral heterogeneity

### The influence of ground stress difference

The in-situ stress under the original geological structure of the reservoir controls the fracture propagation and fracturing effect of hydraulic fracturing. In order to analyze the influence of in-situ stress difference on fracture propagation, the numerical simulation of vertical maximum horizontal in-situ stress and horizontal minimum in-situ stress difference of 3MPa, 5MPa, 7MPa and 9MPa was established, and the mechanism of different in-situ stress difference on the propagation behavior of hydraulic fractures in heterogeneous reservoirs was analyzed.

**Fig 10:**
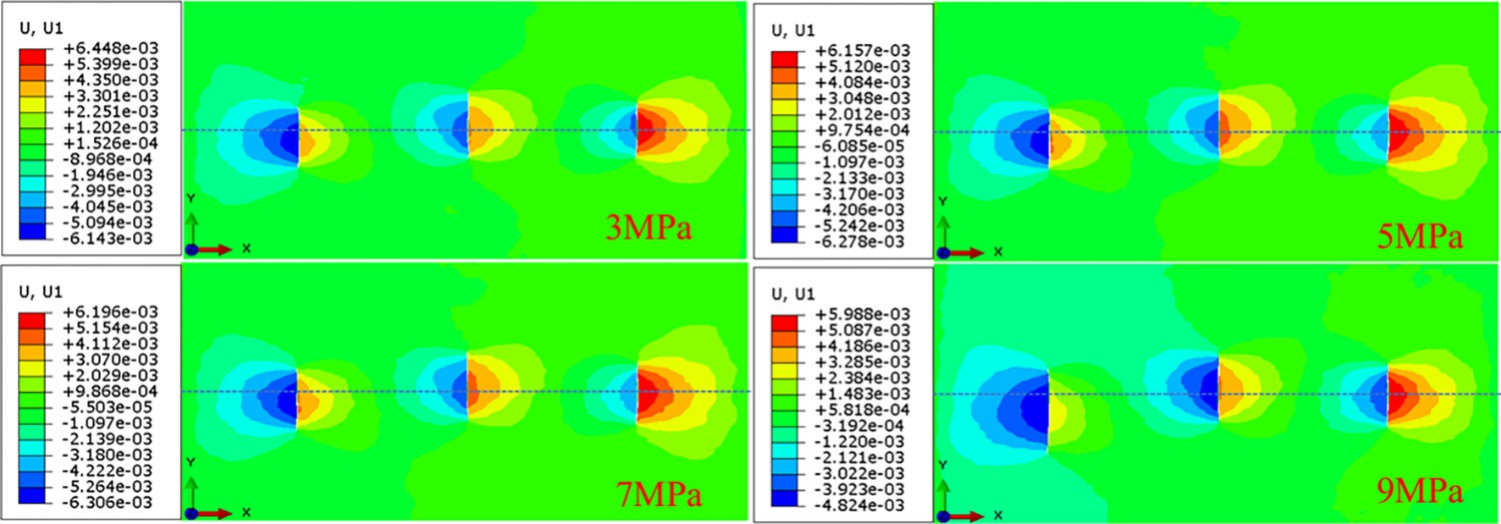
Analysis of influencing factors of hydraulic fracture propagation with different ground stress difference.

As shown in Fig 10, with the decrease of in-situ stress difference, the asymmetric propagation trend of hydraulic fractures is obviously enhanced, and the average fracture width increases. When the in-situ stress differences is small, the three clusters of hydraulic fractures have different degrees of asymmetric propagation. When the in-situ stress differences is 7MPa and 9MPa, there is a cluster of fractures showing asymmetric propagation. This is because when the hydraulic fracture propagates along the direction of the maximum horizontal principal stress in the vertical direction, it is necessary to overcome the squeezing effect of the minimum horizontal ground stress in order to promote its vertical propagation. The greater the in-situ stress difference, the greater the minimum horizontal principal stress to be overcome, which will lead to the decrease of the actual net pressure in the hydraulic fracture, which will lead to the lack of energy for the propagation of the hydraulic fracture, and at the same time, it will be subjected to the non-uniform extrusion of the surrounding strata, which will eventually lead to the emergence of asymmetric propagation morphology. Under the same injection volume, as the ground stress decreases, the fracturing fluid accumulates, the width of the hydraulic fracture is propagated, and the vertical propagation is reduced. When the in-situ stress difference is reduced by 6MPa, the average fracture width of the hydraulic fracture is increased by 48%.

**Fig 11:**
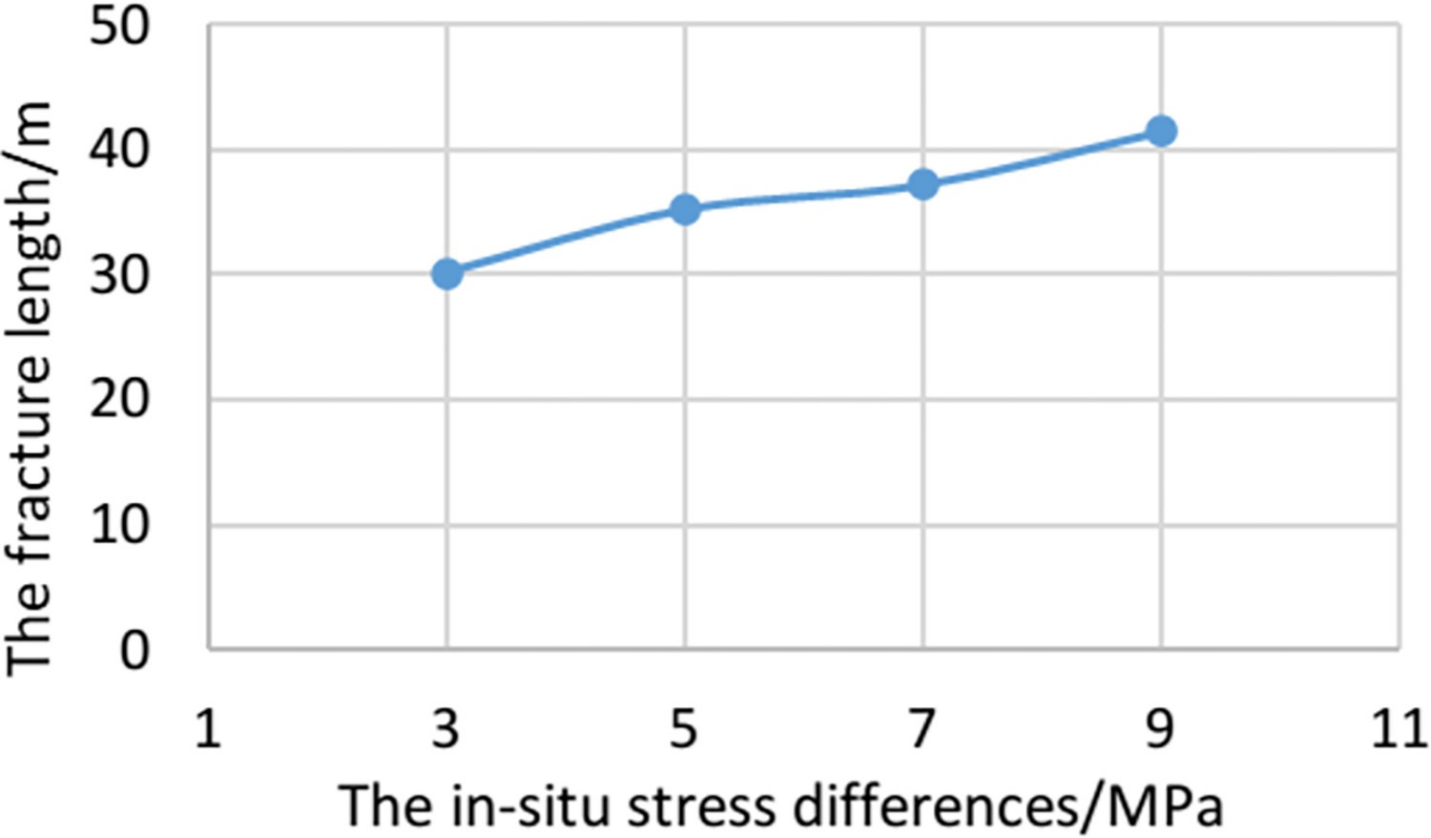
Total fracture length under different ground stress difference.

In order to compare the effect of multi-cluster hydraulic fracture propagation under different stress differences, the secondary development Python code is used to obtain the total length of hydraulic fracture propagation. As the in-situ stress differences increases, the total length of hydraulic fractures increases, as shown in Fig 11. With the increase of stress difference, the length of fracture increases. When the in-situ stress difference increases from 3MPa to 9MPa, the total length of hydraulic fracture increases by 11.4m.

### The influence of injection rate of fracturing fluid

**Fig 12:**
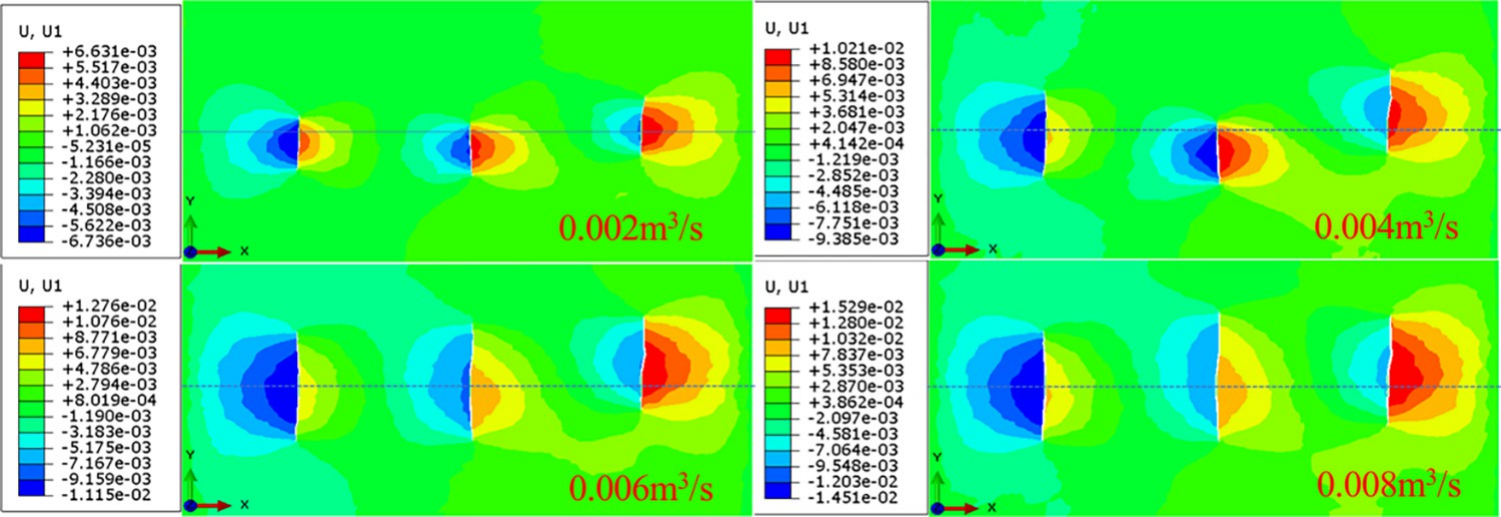
Analysis of influencing factors of hydraulic fracture propagation with different injection rate.

In the multi-fracture propagation of horizontal wells, the injection rate of fracturing fluid is an important factor affecting the fracture propagation morphology, which has an impact on the fractures at different locations. The multi-fracture propagation mainly presents symmetrical propagation and propagation to both sides. In order to analyze the influence of the injection rate of fracturing fluid on the fracture morphology in the heterogeneous model, the fracture propagation model is established when the displacement is 0.002 m^3^/ s, 0.004m^3^/ s, 0.006m^3^/ s and 0.008m^3^ /s, as shown in Fig 12.

Under the condition of the same liquid injection volume, with the increase of the injection rate, the hydraulic fracturing will accelerate the liquid flow rate, which will lead to the increase of the net pressure inside the fracture and the forward propagation, so that the average width of the fracture is reduced accordingly. Increasing the injection rate will increase the flow rate of the fracturing fluid in the hydraulic fracture, thus accelerating the propagation of the hydraulic fracture. In addition, the different characteristics of the formation will also affect the propagation path of the hydraulic fracture and the deformation ability of the rock, resulting in a variety of different hydraulic fractures showing different asymmetric propagation patterns.

**Fig 13:**
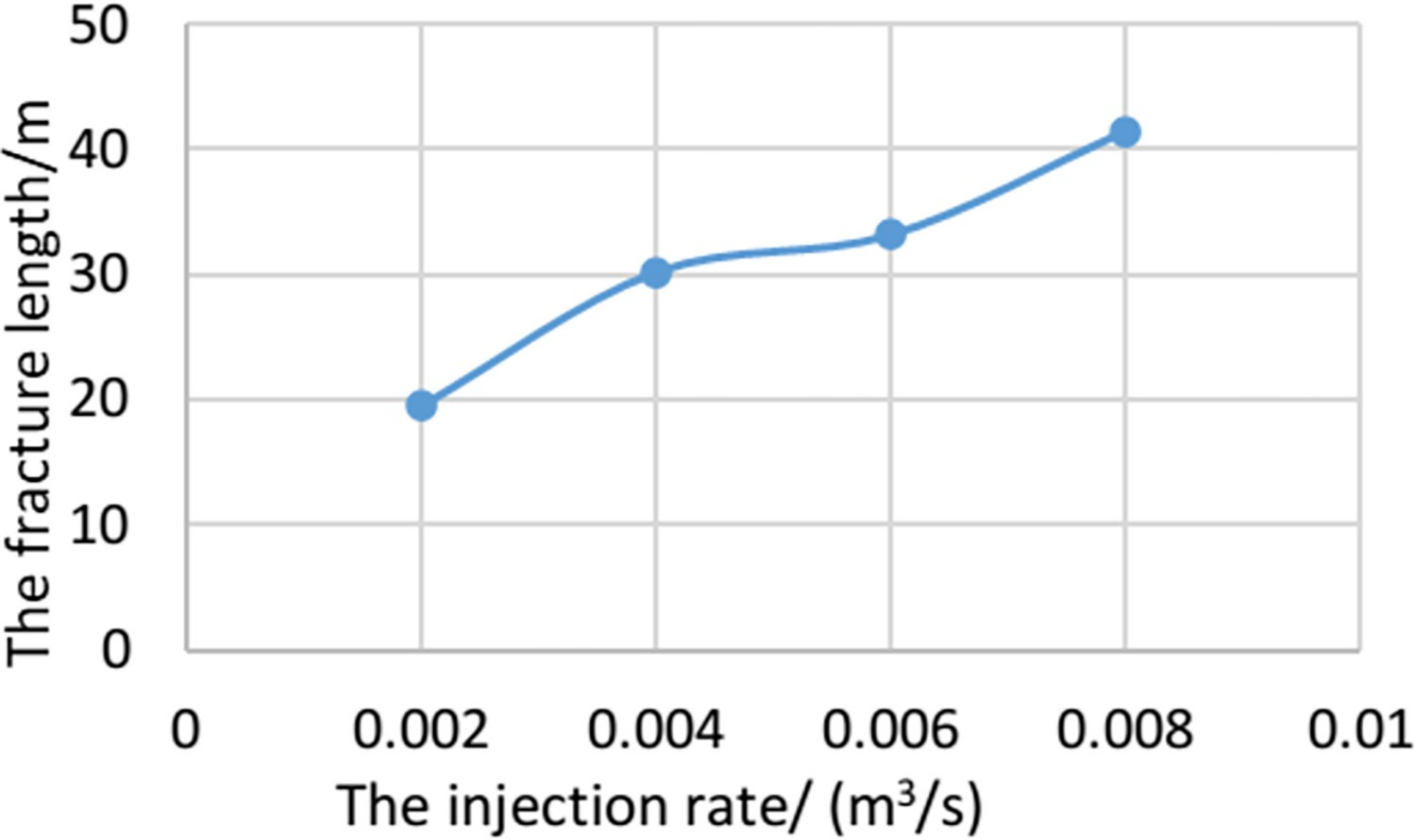
The fracture propagation length under different injection rate.

As shown in Fig 13, when the injection rate is doubled, hydraulic fracturing will cause the average fracture width to decrease by 9.3%. When the injection time is the same, the total length of the hydraulic fracture increases with the increase of the injection rate. When the injection rate increases from 0.002 m^3^ / s to 0.006 m^3^ / s, the total length of hydraulic fractures is 150%. This is because under the condition of the same injection time, the total injection volume of high injection rate is large, which promotes the increase of hydraulic fracture length.

### The influence of fracturing cluster spacing

**Fig 14:**
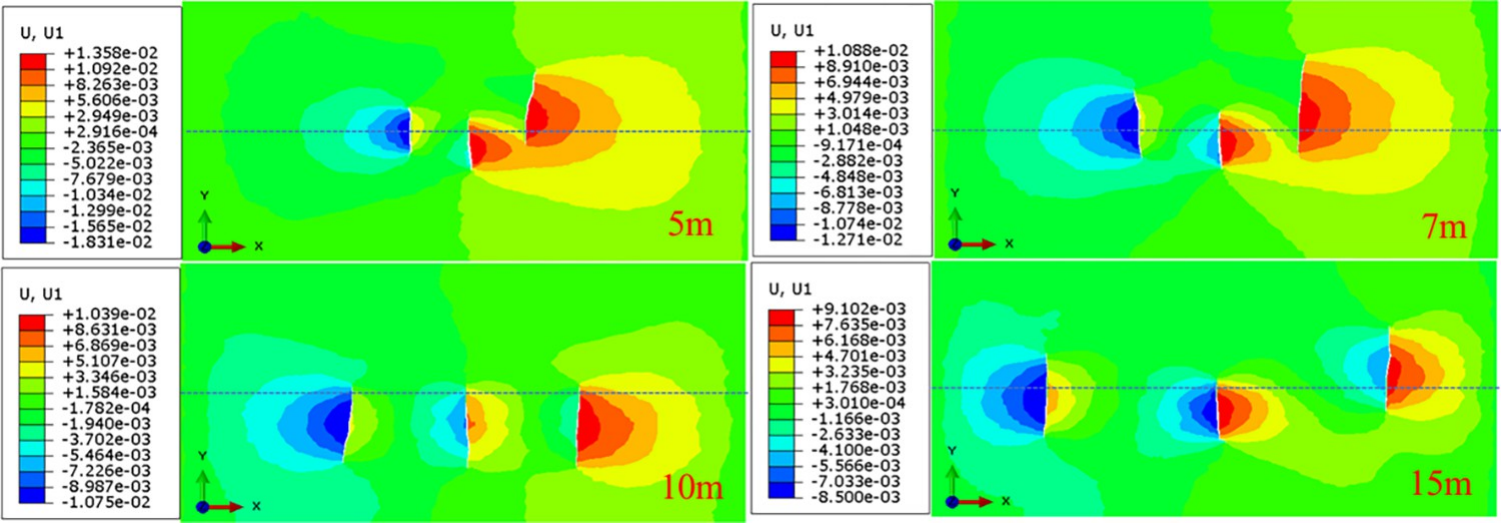
Horizontal displacement diagram of three clusters of hydraulic fracture propagation under different cluster spacing conditions.

When multi-cluster hydraulic fractures propagate, reservoir heterogeneity and stress interference between fractures need to be considered. A multi-fracture propagation model with cluster spacing of 5m, 7m, 10m and 15m was established. Four groups of different clusters spacing were used to characterize the strength of stress interference, analyze the influence on the propagation morphology of three clusters of hydraulic fractures, and reveal the influence of inter-fracture stress interference on the propagation of hydraulic fractures in heterogeneous reservoirs. Drawing the fracture propagation morphology of different cluster spacing, it can be seen that the stress disturbance in the heterogeneous reservoir causes the complex multi-fracture propagation morphology, as shown in Fig 14.

It can be seen from Fig 14 that in the heterogeneous reservoir, the morphological propagation of the three hydraulic fractures has asymmetric performance. When the hydraulic fracture spacing is small (5m), due to the strong inter-fracture stress interference, the fractures on both sides will have different degrees of deflection propagation during the initial fracture propagation. When the spacing of hydraulic fractures increases (7m), the interference between fractures decreases, but it is different from the symmetrical propagation of fractures on both sides of homogeneous reservoirs. When the fracture spacing is 15 meters, the hydraulic fractures on both sides of the fractures begin to propagate in the vertical direction when the initial fractures propagate, and are affected by the heterogeneity, resulting in the propagation of the hydraulic fractures to the area with high elastic modulus and slight deflection propagation. It can be seen that in heterogeneous reservoirs, the effect of fracturing cluster spacing on multi-fracture propagation is different from that of homogeneous reservoirs. In heterogeneous reservoirs, multi-fracture propagation is not symmetrical. At the same time, with the increase of fracture spacing, the influence between fractures is reduced, and fracture propagation is affected by reservoir heterogeneity.

## Conclusion

Heterogeneity has an important impact on rock fracture and fracture propagation. However, the conventional hydraulic fracture propagation model rarely considers heterogeneity. The Weibull distribution method and finite element method were used to establish a hydraulic fracture propagation model considering heterogeneity, and the influence of heterogeneity on rock fracture and fracture propagation was analyzed. The following conclusions were obtained:

1. The characterization model of heterogeneity is established, and the rock breaking law of uniaxial compression experiment with different heterogeneity characteristics is analyzed. It is clear that the rock of heterogeneous reservoir is more likely to fracture at multiple points, and the fracture initiation law is different from that of homogeneous reservoir.
2. A single fracture propagation analysis model of heterogeneous tight sandstone reservoir is established. The sensitivity analysis of heterogeneity characterization methods such as elastic modulus, Poisson ’s ratio and tensile strength is carried out. It is determined that the characterization method of elastic modulus heterogeneity is sensitive to the propagation morphology of hydraulic fractures.
3. Based on the characterization method of elastic modulus heterogeneity, a multi-fracture propagation analysis model of heterogeneous tight sandstone reservoir is established, and the multi-fracture propagation law of heterogeneous reservoir is analyzed. With the increase of the stress difference and the increase of the injection rate, the fracture propagation length increases significantly, and the cluster spacing has a significant effect on the fracture propagation morphology.
4. Heterogeneity characterization model can be applied in many aspects, including borehole stability analysis, shale fracture propagation analysis, etc. Heterogeneity characterization model can be applied in drilling, oil production and other petroleum engineering technologies. Next, the research of horizontal well multistage fracturing propagation, which takes into account both fractures induced heterogeneity and reservoir mineral heterogeneity, will have more extensive applications.

The actual reservoir is heterogeneous, and the research model is simplified as homogeneous reservoir. However, the research of the fracture characteristics and the law of reservoir fracture propagation of homogeneous reservoir has certain errors with the actual rock. This error causes the actual engineering parameters to be high or low, which can not make the best use of the reservoir. The research in this paper is helpful to understand the fracture behavior of actual rock. The research has guiding significance for the design of fracturing and parameter optimization in practical engineering.

## Supporting information

S1 Dataset (RAR)
